# Regulation of microglial polarization via notch signaling pathway by intrathecal administration of tanshinone IIA-PLGA sustained-release microspheres to promote neurological recovery after spinal cord injury

**DOI:** 10.3389/fbioe.2026.1784592

**Published:** 2026-06-05

**Authors:** Chuanhong Li, Luchun Xu, Guozheng Jiang, Kaitan Yang, Wenqing Zhong, Zheng Cao, Xiumei Wang, Yongdong Yang, Xing Yu

**Affiliations:** 1 Department of Orthopedics, The First Affiliated Hospital of Chongqing University of Chinese Medicine (Chongqing Traditional Chinese Medicine Hospital), Chongqing University of Chinese Medicine, Chongqing, China; 2 Department of Orthopedics, Dongzhimen Hospital, Beijing University of Chinese Medicine, Beijing, China; 3 Department of Orthopedics, Hospital Affiliated to Jiangxi University of Chinese Medicine, Jiangxi University of Chinese Medicine, Jiangxi, China; 4 School of Materials Science and Engineering, Tsinghua University, Beijing, China

**Keywords:** microglial polarization, neurological recovery, spinal cord injury, sustained-release microspheres, tanshinone IIA

## Abstract

Secondary injuries following spinal cord injury (SCI) are closely associated with the activation of microglia and neuroinflammation. Tanshinone IIA (TIIA), a traditional Chinese medicine monomer with anti-inflammatory and antioxidant properties, has shown neuroprotective effects after SCI. Our previous studies indicated that TIIA exhibited inhibitory effects on early inflammatory responses and microglial activation after SCI, but its underlying mechanisms remained unclear. Due to TIIA’s poor water solubility and low bioavailability, its sulfonated derivative, sodium tanshinone IIA sulfonate (STS), was developed to address these issues. However, STS still faced challenges in crossing the blood-spinal cord barrier and exhibited poor stability. In this study, we utilized an emulsion electrospinning technique to load TIIA into poly (lactic-co-glycolic) acid (PLGA) microspheres, creating TIIA-loaded PLGA sustained-release microspheres (TIIA-PLGA SRMs). A single injection of an adequate amount of TIIA-PLGA SRMs was administered into the subarachnoid space through the atlanto-occipital membrane, and its regulatory effects on microglia after SCI were compared with a traditional continuous intraperitoneal injection of STS. Our experimental results demonstrated that intrathecal injection of TIIA-PLGA SRMs effectively promoted the transition of microglial phenotypes from M1 to M2 in the early stages in rats after SCI, alleviating neuroinflammation and improving motor dysfunction and neuropathic pain after SCI. Furthermore, the efficacy of TIIA-PLGA SRMs was superior to the traditional continuous intraperitoneal injection of STS. Additionally, our experiments suggested that the mechanism underlying TIIA-PLGA SRMs in regulating microglial polarization after SCI may be associated with the inhibition of the Notch signaling pathway.

## 1 Introduction

Spinal cord injury (SCI) is a highly disabling and fatal disorder of the central nervous system, with increasing incidence and severe consequences (Skinnider et al., 2024; Choi et al., 2023). The pathological mechanisms of SCI involve both primary and secondary injuries, where external trauma directly causes primary injury to the spinal cord. Following primary injury, factors such as inflammation, oxidative stress, and apoptosis contribute to widespread and severe secondary damage, leading to sustained impairment of motor and sensory neural functions (Francos-Quijorna et al., 2022; Fan et al., 2022). Primary injuries are challenging to target for specific treatment due to irreversible damage to the spinal cord’s anatomical structure. As the pathological processes of secondary injuries may persist from weeks to years, there exists a temporal window to intervene and prevent their progression. Therefore, current clinical strategies for the prevention and treatment of SCI focus mainly on mitigating secondary injuries (Jaffer et al., 2023; Ortega et al., 2023).

Microglia, the resident mononuclear phagocytes in spinal cord tissues, play a role in tissue surveillance and injury response (Brockie et al., 2024; Lee et al., 2023). Under physiological conditions, microglia exist in an M0 phenotype, continuously monitoring the spinal cord’s microenvironment through highly active branching processes (Akhmetzyanova et al., 2023; Brockie et al., 2021). After SCI, microglia activate quickly into two phenotypes, M1 and M2. By releasing large amount of oxidative metabolites, inflammatory factors, matrix metalloproteinases, chemokines, and increasing the expression of iNOS, antigen presentation-associated molecules, Toll-like receptors, etc., M1 microglia can enhance the phagocytic ability towards apoptotic cells and promote the formation of protective glial scars, thereby improving the defense, clearance, and limitation capabilities of the central nervous system against harmful stimuli, which is beneficial for maintaining the stability of the neural internal environment. However, at the same time, they still cause neurotoxic effects such as the expansion of neuroinflammation, exacerbation of nerve tissue damage, neuronal death, and excessive formation of glial scars that impede axonal regeneration. Therefore, M1 phenotype is also referred to as the pro-inflammatory or neurotoxic phenotype. In contrast, M2 microglia is characterized by the secretion of neurotrophic factors and anti-inflammatory cytokines, high expression of scavenger receptors and Arg1, and enhanced phagocytic capacity towards damaged myelin sheaths, thereby exerting anti-inflammatory and neurotrophic support effects and increasing the tolerance of nerve tissue to external harmful stimuli. These effects contribute to the reconstruction of the blood-spinal cord barrier (BSCB), inflammation control, restoration of internal environment homeostasis, neural repair and regeneration, and other injury repair processes. Hence, M2 phenotype is also called anti-inflammatory or neuroprotective phenotype (Gu et al., 2023; Fu et al., 2022). In addition, the speed and quality of phagocytosis of M1 and M2 phenotypes are different (Cherry et al., 2014). M1 forms phagosomes with weaker acidity and slower maturation after phagocytosis of necrotic nerve tissue, which is conducive to processing antigens and presenting antigens to T lymphocytes, triggering a series of downstream immune reactions. However, M2 produces rapidly maturing and highly acidic phagosomes, which efficiently clear necrotic harmful substances while creating conditions for the repair of SCI. Therefore, moderating the polarization of microglial phenotypes, inhibiting M1 polarization, and promoting M2 polarization after SCI can alleviate secondary injuries and accelerate functional recovery of the spinal cord.

Tanshinone IIA (TIIA), a lipophilic component of the traditional Chinese herb Danshen (*Salvia miltiorrhiza* Bge.), has been confirmed through modern pharmacological research to possess extensive neuroprotective effects, including anti-inflammatory, antioxidant, and immunomodulatory properties (Sherawat and Mehan, 2023; Subedi and Gaire, 2021). In recent years, TIIA has been gradually applied in SCI treatment with positive outcomes, offering a new direction for SCI treatment (Xu L. et al., 2023). Our previous studies also found that TIIA can promote the recovery of neurological dysfunction after SCI, but its underlying mechanisms need further exploration (Yang et al., 2017). Despite TIIA’s promising prospects, its use is limited by its lipophilic nature, making oral or gastric absorption difficult, resulting in low bioavailability (Jiang et al., 2013). The sulfonated derivative of TIIA, sodium tanshinone IIA sulfonate (STS), significantly improves its water solubility and bioavailability, thus facilitating its formulation in injectable dosage forms. However, the high polarity of STS causes its penetration through the BSCB to be challenging, and its poor stability also poses limitations to its application in clinical and basic research (Qin et al., 2016). Sustained-release microspheres (SRMs) disperse drugs in a high-molecular-weight biopolymer matrix, forming spherical bodies with particle sizes ranging from 5 μm to 250 μm. This approach improves bioavailability and reduces the overall dosage of the drug (Zhang M. et al., 2021). Poly (lactic-co-glycolic acid) (PLGA) is a medically suitable high-molecular-weight polymer with good biodegradability and biocompatibility, making it a suitable carrier for preparing SRMs containing TIIA (Azizi et al., 2020; Zeraatpisheh et al., 2021). Our previous research, utilizing an emulsion electrospinning technique, successfully loaded TIIA into PLGA microspheres, resulting in uniform-sized microspheres with a particle size of 5–10 μm and stable drug release (Yao et al., 2017). This method is suitable for studies on the repair and recovery of SCI.

Intrathecal drug administration after SCI can deliver drugs directly into the subarachnoid space, reaching the lesion site of the spinal cord. This approach avoids limitations of the BSCB and the first-pass elimination effect in the gastrointestinal tract, enhancing drug efficiency (Chen et al., 2021). While traditional intrathecal drug administration methods are mature, issues such as low single injection volume, long-term placement of intrathecal catheters, and multiple puncture administrations still remain (Bang et al., 2018). Furthermore, in our preliminary experiments, we observed that the limited subarachnoid space in conventional intrathecal administration poses challenges in executing a single large-dose intrathecal injection. However, the intrathecal injection via the atlanto-occipital membrane approach allows for the safe administration of a larger dose of TIIA-SRMs in a single injection, thereby optimizing the sustained-release properties of TIIA-SRMs.

Therefore, in this study, we administered a single subarachnoid injection of TIIA-PLGA SRMs via the atlanto-occipital membrane and observed their effects on microglial polarization in SCI rats. Furthermore, we explored the underlying mechanisms of TIIA-PLGA SRMs in exerting neuroprotective effects.

## 2 Materials and methods

### 2.1 Preparation and characterization of TIIA-PLGA SRMs

The TIIA-PLGA SRMs were prepared using emulsion electrospinning method (Yao et al., 2017). A mixture of 10 mg TIIA, 120 mg PLGA, and 2 mL dichloromethane was magnetically stirred for 10 min until complete dissolution of PLGA particles. The resulting drug solution was drawn into a 5 mL syringe and fixed in the microsyringe pusher of the electrospinning device (TEADFS-100, TechNova, Beijing, China). The instrument parameters were set as follows: microsyringe pushing speed at 1 mL/h, high-voltage power supply 1 (positive) at 6.5 kV, high-voltage power supply 2 (negative) at −1.3 kV, temperature at 25 °C, and humidity at 7%. After electrospinning, the receiving plate was placed in a dark and wind-sheltered environment for 30 min. After complete evaporation of chloroform, the microspheres were collected, irradiated with cobalt-60 gamma rays for sterilization, and stored in the dark at 4 °C.

### 2.2 Morphological observation and drug release performance of TIIA-PLGA SRMs

After gold sputter coating, the morphology and size of TIIA-PLGA SRMs were observed using a scanning electron microscope (SEM, Merlin Compact, Zeiss, Germany). The *in vitro* release test of TIIA-PLGA SRMs was conducted using dynamic dialysis, and the content of TIIA was determined by high-performance liquid chromatography (HPLC). The cumulative release percentage Qn of the drug at different time points (6 h, 12 h, 1 day, 2 days, 3 days, 6 days, 9 days, 12 days, 15 days, 18 days, 21 days, 24 days, 27 days, and 30 days) was calculated according to the formula provided below.

$$E=\frac{V_{E}\sum_{1}^{n-1}\;c_{i}+v_{0}C_{n}}{m_{0}}\times 100\%$$

### 2.3 Animals

**FIGURE 1:**
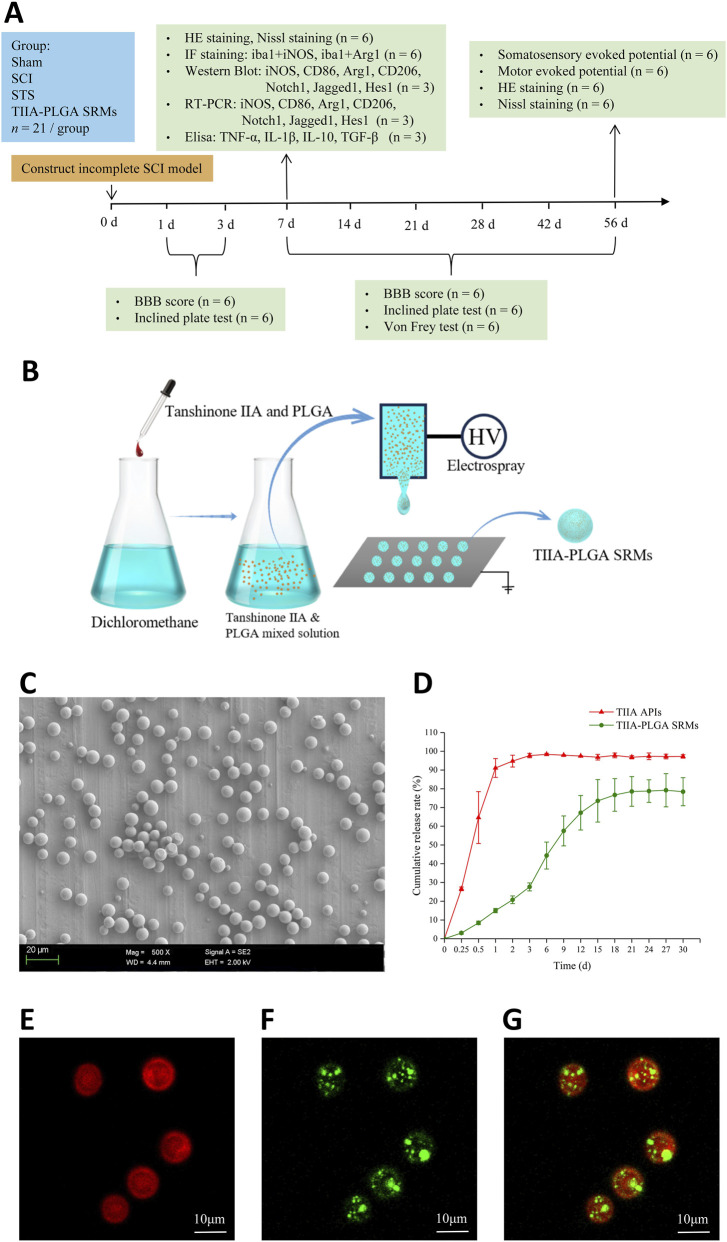
Experimental design, morphology, sustained release performance, and distribution of model drug in TIIA SRMs. **(A)** Experimental design. **(B)** The synthetic route of TIIA-PLGA SRMs. **(C)** Morphology of TIIA-PLGA SRMs observed under scanning electron microscopy. **(D)** *In vitro* cumulative release curve of TIIA-PLGA SRMs. **(E)** The rhodamine labeled the shell of PLGA microspheres; **(F)** The BSA-FITC model drug as multiple nanoscale droplets distributed in the PLGA microspheres; **(G)** The merged image of **(E,F)**, showing the drug-nanoencapsulated PLGA microspheres.

A total of 84 female adult Sprague–Dawley (SD) rats weighing 220–250 g were used in this study. The ethics committee of the Institute of Basic Theory for Chinese Medicine, China Academy of Chinese Medical Sciences (Beijing, China), approved all experimental and animal procedures in this study (Approval No.: 181,278). The experiments were conducted according to the principles outlined in the Guide for the Care and Use of Laboratory Animals by the National Institutes of Health (Bethesda, MD, United States). The rats were housed in a controlled environment with a 12:12 h light-dark cycle, and had unrestricted access to food and water. Each rat was assigned a numbered ear tag. The rats were randomly divided into four groups: sham surgery group (*n* = 21), SCI group (*n* = 21), TIIA-PLGA SRMs group (*n* = 21), and STS group (*n* = 21). A detailed experimental flowchart is provided in Figure 1A to illustrate the overall study design and procedure. The rats were anesthetized with pentobarbital sodium (4% pentobarbital sodium solution 0.1 mL/100 g, i.e., pentobarbital sodium 40 mg/kg, intraperitoneal injection). Subsequently, a laminectomy was performed at the T9 segment to expose the spinal cord. An incomplete SCI animal model (height: 25 mm, weight: 10 g) was established using an NYU impactor (model-II manual, NYU, United States). The sham surgery group underwent laminectomy without SCI.

### 2.4 Drug treatment

After wound closure, drug treatment was administered immediately through a one-time injection into the cisterna magna. In summary, the rats were placed in the prone position with their heads bent forward by 30°. The occipital bone, cisterna magna membrane, and posterior arch of the atlas were fully exposed. A sharp puncture needle was vertically inserted into the center of the cisterna magna. After piercing, the needle was advanced further by 1 mm gradually. The dura mater external catheter, syringe, and microinjection pump were then connected in sequence. Cerebrospinal fluid (100 μL) was slowly drawn at a constant rate. The blunt needle injection needle was then replaced, and the TIIA-PLGA SRMs suspension (40 μL/100 g) was slowly injected into the cisterna magna at a rate of 100 μL/min in TIIA-PLGA SRMs group. The sham surgery group, the SCI group, and the STS group received intrathecal injection of normal saline (40 μL/100 g) in the same manner immediately after surgery. The STS group was started on daily intraperitoneal injections of sodium tanshinone IIA sulfonate injection (20 mg/kg) (provided by SPH No.1 Biochemical & Pharmaceutical Co., Ltd.; Shanghai, China) immediately after surgery, over a period of 7 days. The sham surgery group, the SCI group, and the TIIA-PLGA SRMs group received intraperitoneal injection of normal saline immediately after surgery over a period of 7 days.

### 2.5 Behavioral evaluations

#### 2.5.1 Basso, beattie, bresnahan locomotor rating scale

The Basso, Beattie, and Bresnahan (BBB) locomotor rating scale was used to assess hind limb motor function (Basso et al., 1995). The scale evaluates stepping ability, bilateral joint movement, coordination, and trunk stability, with higher scores indicating better motor function. Assessments were conducted before injury and at 1, 3, 7, 14, 21, 28, 42, and 56 days post-injury in an open field, with 6 rats from each group being assessed at each time point.

#### 2.5.2 Inclined plate test

According to previous reports (Rivlin and Tator, 1977), a rough-surfaced rectangular wooden board was prepared. Rats were positioned on the board with their heads against the wall, and the longitudinal axis of their bodies parallel to the long side of the board. The board was then raised against the wall, and the angle formed between the long side of the board and the experimental operating table was measured with a goniometer. The result was recorded as the maximum angle at which the rat could stay on the inclined board for at least 5 s. Assessments were performed before injury and at 1, 3, 7, 14, 21, 28, 42, and 56 days post-injury, with 6 rats from each group being assessed at each time point.

#### 2.5.3 Von frey test

Mechanical pain threshold was assessed using Von Frey hairs, as previously described (Cheng et al., 2023). Von Frey hairs (NC12775-99, North Coast Medical Inc., San Jose, CA, United States) were applied in ascending force order, and the median withdrawal threshold was calculated based on values after one descent and two ascents. Evaluations were conducted before injury and at 7, 14, 21, 28, 42, and 56 days post-injury, with 6 rats from each group being assessed at each time point.

### 2.6 Neurophysiological examination

#### 2.6.1 Somatosensory evoked potential (SEP)

Conducted at 8 weeks post-injury, the rats were first anesthetized with pentobarbital sodium (with the former method). The stimulating needle electrode was fixed near the posterior tibial nerve in the right hind limb, the reference electrode was placed subcutaneously at 1 mm from the stimulating electrode, and the grounding electrode was placed subcutaneously at the back. Single-pulse square wave stimulation was applied to the tibial nerve (intensity: 3.5 mA, frequency: 2 Hz, pulse width: 0.1 m, overlay stimulation frequency: 400 times), and the latency and amplitude of SEP were observed and recorded. The amplitude was the potential difference between the peak P40 of the specific SEP waveform and the baseline represented by P40’. Six rats from each group were assessed during this procedure.

#### 2.6.2 Motor evoked potential (MEP)

Performed at 8 weeks post-injury, the rats were first anesthetized with pentobarbital sodium (with the former method). The needle electrode was placed subcutaneously on the top of the head, the reference electrode was placed subcutaneously at 1 mm from the stimulating electrode, and the grounding electrode was placed subcutaneously in the gastrocnemius muscle of the right hind limb. Single-pulse square wave stimulation was applied to the cerebral cortex (intensity: 45 mA, frequency: 1 Hz, pulse width: 0.1 m), and the peak-to-peak amplitude (P–P amplitude) and latency of MEP were observed and recorded. The P–P amplitude was the potential difference between adjacent peaks and troughs of the specific MEP waveform. Six rats from each group were assessed during this procedure.

### 2.7 Tissue processing and histopathology

For each group, 12 rats were perfused with physiological saline and subsequently perfused with 4% paraformaldehyde (Beijing Labgic Technology Co. Ltd., Beijing, China) after euthanasia with narcotic overdose (4% pentobarbital sodium solution 0.3 mL/100 g, i.e., pentobarbital sodium 120 mg/kg, intraperitoneal injection). After perfusion, a 2 cm-long spinal cord tissue sample was taken from the center of the injury site (T9), placed in a 30% sucrose solution (Cat# CS10581, Beijing Coollaibo Technology Co., Ltd. Beijing, China) for dehydration, embedded in optimal cutting temperature compound and longitudinal frozen sections in sagittal plane were performed on a cryostat (Leica Camera AG, Weztlar, Germany) at a thickness of 10 μm for hematoxylin and eosin (HE) staining, 12 μm for Nissl staining, and 10 μm for immunofluorescence staining. The remaining rats were euthanized without perfusion, and the spinal cord was extracted for further analysis. Frozen sections and tissues were stored at −80 °C.

### 2.8 HE staining

HE staining was performed at 1 and 8 weeks post-SCI. Frozen sections stained with HE were dehydrated with a gradient of ethanol, cleared with xylene for transparency, and then sealed in neutral gum. Digital slide scanner (3DHISTECH Ltd., Budapest, Hungary) was used to capture images. The severity of pathological morphology at 1 week post-injury was scored according to the SCI severity pathological score (Yin et al., 2012). Evaluation criteria included: neural tissue edema, immune/inflammatory cell infiltration, bleeding foci, and disorderly neural tissue structure, with scores ranging from 0 to 4; 0 for no or slight damage, 1 for mild or limited damage, 2 for moderate damage, 3 for widespread or severe damage, and 4 for widespread and particularly severe damage. At 8 weeks post-injury, the transverse diameter of the SCI site was quantitatively evaluated to assess the degree of injury and repair. The transverse diameter of the SCI site was calculated as follows (minimum transverse diameter of the SCI site (excluding scar tissue and intraspinal cord cavities)/average maximum transverse diameter of the normal spinal cord at the head and tail ends) × 100%. Six rats from each group were assessed during this procedure.

### 2.9 Nissl staining

Nissl staining was performed at 1 and 8 weeks post-SCI. Frozen sections were immersed in Nissl staining solution (cresyl violet method, Cat# G1430, Solarbio Life Science Co., Ltd., Beijing, China) at 56 °C for 60 min. After staining, differentiation was performed for 1 min, followed by dehydration in 100% ethanol, immersion in xylene for transparency, and then sealed in neutral gum. Digital slide scanner (3DHISTECH Ltd.) was used to capture images. Six rats from each group were assessed during this procedure.

### 2.10 Immunofluorescence staining

Immunofluorescence staining was performed at 1 week post-SCI. Frozen sections were immersed in citrate antigen retrieval solution (Cat# MVS-0066, Fuzhou MaiXin Biotechnology Development Co., Ltd., Fuzhou, China) and heated for 15 min, followed by incubation at 22–25 °C for 10 min with a non-specific staining blocker (Cat# KIT-9701, Fuzhou MaiXin Biotechnology Development Co., Ltd.). Primary antibodies, namely, Iba1 (1:50, Cat# sc-32725, Santa Cruz Biotechnology, Inc., Santa Cruz, CA, United States), iNOS (1:100, Cat# 18985-1-AP, Proteintech Group, Inc., Chicago, IL, United States), and Arg1 (1:100, Cat# ab96183, Abcam plc., Cambridge, United Kingdom) were then incubated with the sections overnight at 4 °C. Subsequently, secondary antibodies, namely, goat anti-mouse IgG (1:200, Cat# ab150117, Abcam plc.) and goat anti-rabbit IgG (1:200, Cat# ab150084, Abcam plc.), were incubated with the sections in a humid chamber for 1 h. After washing, the sections were stained with 4′,6-diamidino-2-phenylindole (DAPI, Cat# ab104139, Abcam plc.) sealing agent. Digital slide scanner was used to capture images, and semi-quantitative analysis of the expression of M1 and M2 microglial cell markers (M1: iNOS, M2: Arg1) was performed. Five random fields (×200) were selected per section, and the mean grayscale value of red fluorescence (iNOS or Arg1), representing the mean fluorescence intensity, was measured using ImageJ software (National Institutes of Health). Simultaneously, the number of positively immunostained cells in the lesion area was quantitatively analyzed. Specifically, the number of Iba1^+^ + iNOS^+^ (M1 microglia) and Iba1^+^ + Arg1^+^ (M2 microglia) cells was counted, and these were co-localized with DAPI^+^ nuclei to confirm cell identity, the results were normalized to the proportion of all microglia (Iba1^+^). The fluorescence intensity data were normalized using the background fluorescence intensity to reduce variability between sections. Specifically, the mean background fluorescence was subtracted from the measured intensity in each field. This adjustment minimized differences caused by staining or imaging conditions. Six rats from each group were assessed during this procedure.

### 2.11 Real-time polymerase chain reaction

Real-time Polymerase Chain Reaction (RT-PCR) was performed at 1 week post-SCI. Total RNA was extracted from the injured spinal cord using TRIzol reagent (Invitrogen, United States) (3 samples per group). The extracted RNA (1 μg) was reverse transcribed into cDNA using the M-MLV RT kit (Promega, United States). RT-PCR was performed using a Real-Time PCR kit (Qiagen, Germany). The reaction system consisted of 2 μL of cDNA, 10 μL of SYBR mix, and 0.5 μL of each primer. The PCR conditions were as follows: preincubation at 94 °C for 2 min, followed by 40 cycles of denaturation at 94 °C for 5 s, and annealing/extension at 60 °C for 30 s using the Exicycler™ 96 detection system. Relative gene expression levels were quantified using the 2^−ΔΔCT^ method, with GAPDH as an internal control. Three rats from each group were assessed during this procedure. The primer details are provided in Table 1 (iNOS, CD86, Arg1, CD206, Notch1, Jagged1, and Hes1).

**TABLE 1:** Primers of RT-PCR used in this study.

Target	Forward (5′-3′)	Reverse (5′-3′)
iNOS	TTC​ATG​AAG​CAC​ATG​CAG​AA	ACA​TCT​CCT​GGT​GGA​ACA​CA
CD86	TGT​CTC​TTT​CTG​CTG​GTC​GT	ATC​GAC​TCG​TCA​ACA​CCA​CT
Arg1	TTG​TGA​AGA​ACC​CAC​GGT​CT	AAT​GAC​GCA​TAG​GTC​AGG​GT
CD206	TGG​GCT​TAT​GGA​GAG​CCA​AA	TCT​GCA​GTC​ACT​GGT​GGA​TT
Notch1	ATG​GCT​CCA​TCG​TCT​ACC​TG	TAC​ATG​AGG​TGC​AGC​TGT​GA
Jagged1	ATA​CAC​GTG​GCC​ATT​TCT​GC	GCT​GTT​CCC​ATC​CCG​TTT​AC
Hes1	CAA​AGA​CAG​CCT​CTG​AGC​AC	ATG​TCT​GCC​TTC​TCC​AGC​TT
GAPDH	CAA​CTC​CCT​CAA​GAT​TGT​CAG​CAA	GGC​ATG​GAC​TGT​GGT​CAT​GA

### 2.12 Western blot analysis

Western blot analysis was performed at 1 week post-SCI. Proteins were extracted according to the instructions of the Protein Extraction Kit (Cat# GPP 1815, Beijing Genepool Biotechnology Co., Ltd., Beijing, China). A total of 80 μg of protein was added to sodium dodecyl sulfate-polyacrylamide gel electrophoresis (SDS-PAGE) loading buffer (5×, Cat# GPP 1820, Beijing Genepool Biotechnology Co., Ltd.) and pure water to appropriate concentration, and then boiled for protein denaturation. Equal amounts of proteins were separated by electrophoresis on a 6%–12% SDS PAGE gel. The proteins were then transferred to a polyvinylidene fluoride membrane (0.22 μm pore size), and the membrane was blocked with milk blocking buffer. Subsequently, the membrane was incubated overnight at 4 °C with the following primary antibodies: iNOS (1:1,000, Cat# 18985-1-AP, Proteintech Group, Inc.), CD86 (1:1,000, Cat# 82882-1-RR, Proteintech Group, Inc.), Arg1 (1:2000, Cat# 16001-1-AP, Proteintech Group, Inc.), CD206 (1:2000, Cat# 18704-1-AP, Proteintech Group, Inc.), Notch1 (1:2000, Cat# 10062-2-AP, Proteintech Group, Inc.), Jagged1 (1:2000, Cat# ab300561, Abcam plc., Cambridge, United Kingdom), and Hes1 (1:2000, Cat# ab108937, Abcam plc., Cambridge, United Kingdom). After incubation, the membrane was washed and secondary antibodies were added and incubated at 22–25 °C for 50 min. After which, the membrane was washed again, and immersed in enhanced chemiluminescence (ECL) color development solution for 1 min, followed by exposure, imaging, and fixing in a darkroom. Finally, ImageJ software (National Institutes of Health) was used to quantify the protein expression in each band. Three rats from each group were assessed during this procedure.

### 2.13 Enzyme-linked immunosorbent assay (ELISA)

ELISA was performed at 1 week post-SCI to detect the expression levels of tumor necrosis factor (TNF)-α, interleukin (IL)-1β, IL-10, and transforming growth factor (TGF)-β cytokines in the injured spinal cord tissue. All ELISA kits were purchased from Shanghai Elisa Biotechnology Inc., Shanghai, China). After grinding the injured spinal cord tissue in normal saline, the supernatant was collected after centrifugation at 3,000 *g* for 10 min. According to the instructions of the ELISA kit, the supernatant was reacted with TNF-α, IL-1β, IL-10, and TGF-β working solutions for 15 min. The absorbance values were determined at 450 nm using an ELISA reader (ELx800, BioTek Instruments, Inc., Winooski, VT, United States). Three rats from each group were assessed during this procedure.

### 2.14 Statistical analysis

Statistical analysis and data visualization were performed using SPSS 26.0 software (IBM Corp., Armonk, NY, United States) and GraphPad Prism 8.0 software (GraphPad software Inc., San Diego, CA, United States). Continuous variables were expressed as means ± S.D. The normality and homogeneity of variance were checked to ensure that the data followed a normal distribution and showed homogeneity of variance. Subsequently, one-way analysis of variance (ANOVA) was used for multiple comparisons, and *post hoc* analysis was performed using least significant difference (LSD) multiple comparison test under the conditions of normal distribution and homogeneity of variance. For continuous data that did not satisfy the requirements of normality or exhibited heterogeneous variances, the Kruskal–Wallis test was utilized. A significance level of *P* < 0.05 was considered statistically significant.

## 3 Results

### 3.1 Morphology and sustained release performance of TIIA-PLGA SRMs

The synthetic route of TIIA-PLGA SRMs is shown in Figure 1B. Under scanning electron microscopy, TIIA-PLGA SRMs were observed to have a well-defined spherical shape with a smooth surface and uniform particle size (Figure 1C). As shown in Figure 1D, the release of TIIA raw material was extremely rapid, reaching an accumulated release rate of over 90% within 1 day of the experiment. In comparison, TIIA-PLGA SRMs exhibited slow release of TIIA in the release medium, with a slightly faster release in the first 15 days, followed by a gradual slowdown. The accumulated release rate stabilized at around 80% after 21 days of the experiment. The entire release process of TIIA-PLGA SRMs was stable, showing no burst release, indicating good sustained release performance. To further investigate the distribution pattern of TIIA within PLGA microspheres, the lipophilic compound BSA-FITC was used as a model drug, and sustained-release microspheres were prepared under the same conditions. Confocal laser scanning microscopy showed that the model drug was evenly distributed within the microspheres (Figures 1E–G).

### 3.2 TIIA-PLGA SRMs intrathecal administration promotes motor function recovery after SCI

**FIGURE 2:**
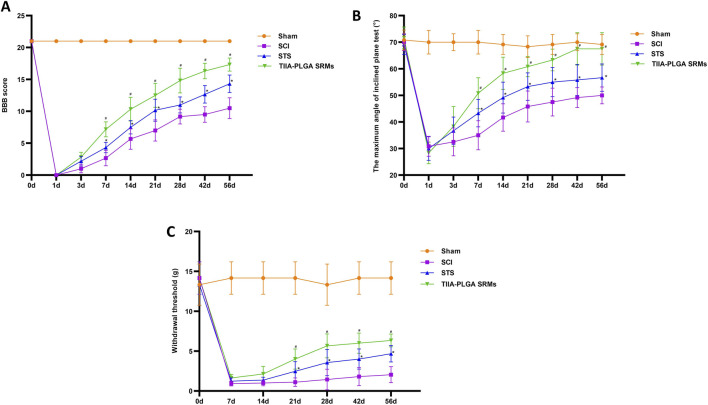
Evaluation of motor and sensory functions in rats after SCI. **(A)** BBB scores; **(B)** Inclined plate test; **(C)** Von Frey test. Data are presented as means ± S.D. (*n* = 6 per group). *P < 0.05 vs. SCI group. #*P* < 0.05 vs. STS group.

The recovery of motor function in rats was assessed at various time points before and after SCI using the BBB score and inclined plate test. As shown in Figure 2A, except for the sham surgery group, all rats exhibited severe motor deficits after surgery. From day 7 to day 56 after injury, the BBB scores of the STS group were significantly higher than those of the SCI group (*P* < 0.05), and the BBB scores of the TIIA-PLGA SRMs group were further significantly increased compared with the STS group (*P* < 0.05). Similarly, the inclined plate test showed a similar trend (Figure 2B), and at 42 and 56 days after injury, the TIIA-PLGA SRMs group had similar results to the sham surgery group (*P* > 0.05). These results indicate that intrathecal administration of TIIA-PLGA SRMs significantly improved the motor function of SCI rats, and the effect is superior to intraperitoneal injection of STS.

### 3.3 TIIA-PLGA SRMs intrathecal administration improves neuropathic pain after SCI

Neuropathic pain in SCI rats was evaluated using the Von Frey test. At 7 days post-SCI, all rats, except in the sham surgery group, began to exhibit mechanical hypersensitivity (Figure 2C). Compared with the SCI group, the STS group showed a significant alleviation of mechanical hyperalgesia in SCI rats (*P* < 0.05), and the TIIA-PLGA SRMs group further significantly improved the relief of neuropathic pain compared with the STS group (*P* < 0.05) (Figure 2C). These results suggest that intrathecal administration of TIIA-PLGA SRMs significantly improved neuropathic pain in SCI rats, and this treatment effect is superior to STS intraperitoneal injection.

### 3.4 TIIA-PLGA SRMs intrathecal administration improves neuroconduction function of injured spinal cord

**FIGURE 3:**
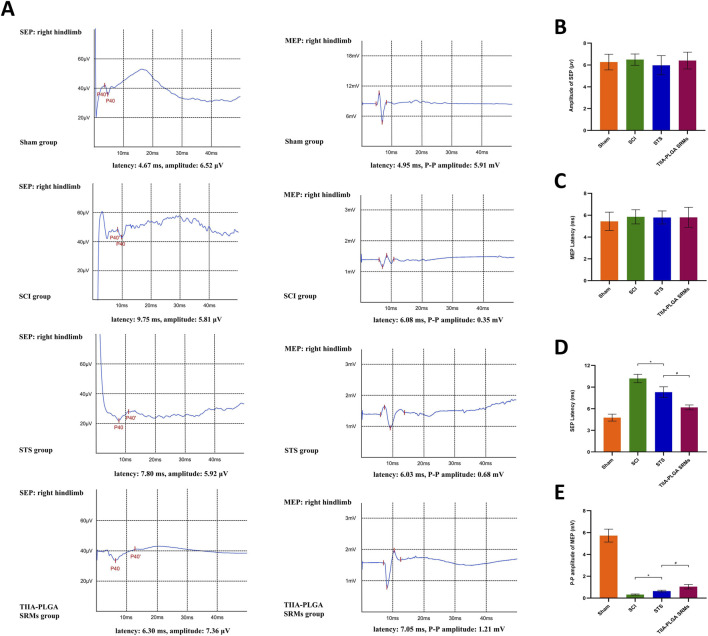
Examination of spinal cord neuroconduction function in rats after SCI. **(A)** Representative waveforms of SEP and MEP; **(B)** Amplitude of SEP; **(C)** Latency of MEP; **(D)** Latency of SEP; **(E)** P–P amplitude of MEP. Data are expressed as the means ± S.D. (*n* = 6 per group). *P < 0.05 vs. SCI group. #*P* < 0.05 vs. STS group.

At 8 weeks post-surgery, SEPs and MEPs waveforms were obtained for each group (Figure 3A). There were no statistically significant differences between the groups in terms of SEP amplitude and MEP latency (Figures 3B,C). Regarding SEP latency, all experimental groups showed a significant increase compared with the sham surgery group (*P*< 0.05). The SEP latency of the STS group was significantly lower than that of the SCI group (*P* < 0.05), and the SEP latency of the TIIA-PLGA SRMs group was further significantly reduced compared with the STS group (*P* < 0.05) (Figure 3D). In addition, in terms of MEP P-P amplitude, all experimental groups showed a significant decrease compared with the sham surgery group (*P*< 0.05). The MEP P-P amplitude of the STS group was significantly higher than that of the SCI group (*P* < 0.05), and the MEP P-P amplitude of the TIIA-PLGA SRMs group was further significantly increased compared with the STS group (*P* < 0.05) (Figure 3E). These results indicate that intrathecal administration of TIIA-PLGA SRMs effectively improved the neuroconduction function of SCI rats, and this treatment effect is superior to STS intraperitoneal injection.

### 3.5 TIIA-PLGA SRMs intrathecal administration improves the pathomorphological manifestations of injured spinal cord

**FIGURE 4:**
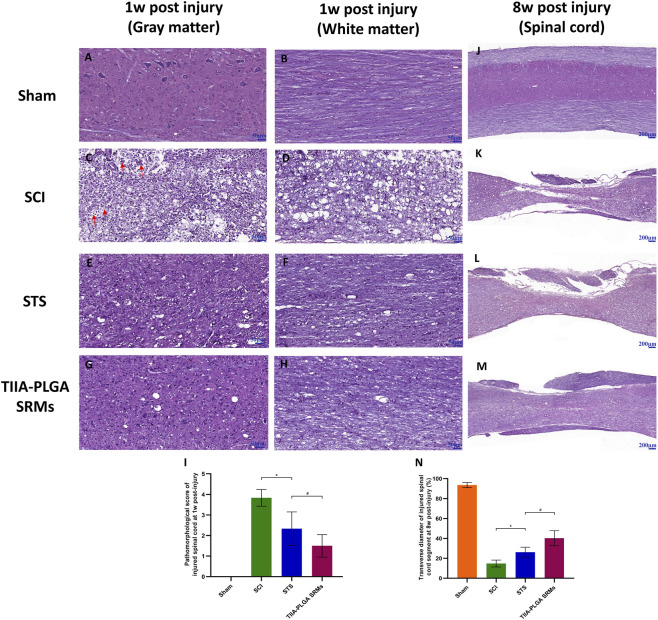
Histological results of spinal cord tissue at the injury site in rats after SCI. **(A)** Gray matter at 1 week post-injury in the sham surgery group; **(B)** White matter at 1 week post-injury in the sham surgery group; **(C)** Gray matter at 1 week post-injury in the SCI group, with red arrows indicating infiltration of inflammatory cells; **(D)** White matter at 1 week post-injury in the SCI group; **(E)** Gray matter at 1 week post-injury in the STS group; **(F)** White matter at 1 week post-injury in the STS group; **(G)** Gray matter at 1 week post-injury in the TIIA-PLGA SRMs group; **(H)** White matter at 1 week post-injury in the TIIA-PLGA SRMs group. Scale bar: 50 μm. **(I)** Pathological morphology scores at 1 week post-injury for each group. Data are expressed as the means ± S.D. (*n* = 6 per group). *P < 0.05, vs. SCI group; #*P* < 0.05 vs. STS group. **(J)** Spinal cord at 8 weeks post-injury in the sham surgery group; **(K)** Spinal cord at 8 weeks post-injury in the SCI group; **(L)** Spinal cord at 8 weeks post-injury in the STS group; **(M)** Spinal cord at 8 weeks post-injury in the TIIA-PLGA SRMs group. Scale bar: 200 μm. **(N)** Transverse diameter of the injury site in each group at 8 weeks post-injury. Data are expressed as the means ± S.D. (*n* = 6 per group). *P < 0.05, vs. SCI group; #*P* < 0.05 vs. STS group.

HE staining showed that at 1 week post-surgery, the sham surgery group displayed scattered normal neurons in the gray matter (Figure 4A), and well-organized nerve fiber bundles without swelling or deformation in the white matter (Figure 4B). In contrast, the SCI group showed severe edema with loose and disordered structures in the gray matter; numerous vacuoles were formed after neuronal death, and widespread infiltration of immune/inflammatory cells were present (Figure 4C). The white matter showed irregular and disordered arrangement of nerve fiber bundles, with “honeycomb-like” changes due to the presence of numerous vacuoles (Figure 4D). Compared with the SCI group, the STS group showed denser gray matter structure with reduced edema and fewer internal vacuoles at 1 week post-surgery. The white matter showed decreased infiltration of immune/inflammatory cells, and more regular arrangement of nerve fiber bundles with fewer vacuoles Figures 4E,F), resulting in a significantly lower pathological morphology score (*P* < 0.05) (Figure 4I). The TIIA-PLGA SRMs group exhibited slight edema of the gray matter at 1 week post-surgery, showing tissue structures similar to the sham surgery group, with scattered vacuoles and swollen neurons (Figure 4G). The arrangement of nerve fiber bundles in the white matter was generally regular, with scattered vacuoles (Figure 4H), resulting in a further significant decrease in the pathological morphology score compared with the STS group (*P* < 0.05) (Figure 4I). At 8 weeks post-surgery, the sham surgery group maintained overall regular spinal cord morphology without atrophy (Figure 4J). The SCI group had severe spinal cord atrophy that was wrapped with scar tissues externally, and elongated cavities were seen within the spinal cord (Figure 4K). The STS group exhibited obvious spinal cord atrophy and internal cavities at 8 weeks, and the transverse diameter of the injury site increased significantly compared with the SCI group (*P* < 0.05) (Figures 4L,N). The TIIA-PLGA SRMs group also showed significant spinal cord atrophy and internal cavities at 8 weeks, but the transverse diameter of the injury site increased more significantly than the STS group (*P* < 0.05) (Figures 4M,N).

**FIGURE 5:**
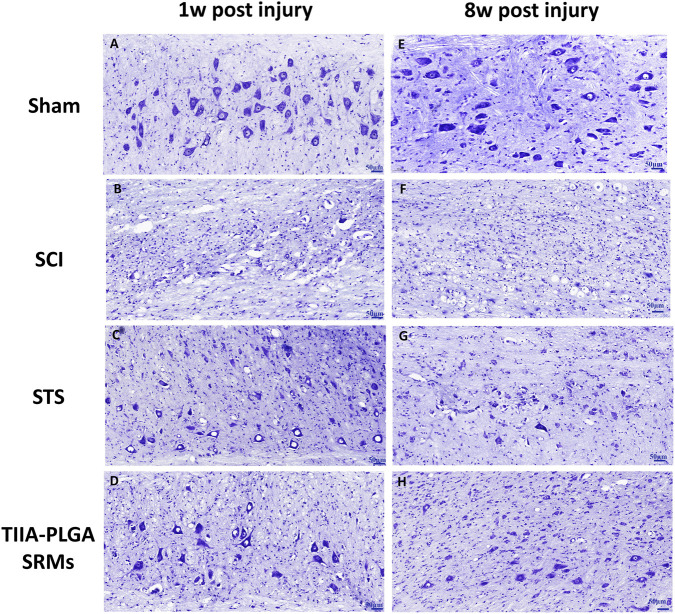
Nissl staining results of spinal cord tissue at the injury site in rats after SCI. **(A)** 1 week post-injury in the sham surgery group; **(B)** 1 week post-injury in the SCI group; **(C)** 1 week post-injury in the STS group; **(D)** 1 week post-injury in the TIIA-PLGA SRMs group; **(E)** 8 weeks post-injury in the sham surgery group; **(F)** 8 weeks post-injury in the SCI group; **(G)** 8 weeks post-injury in the STS group; **(H)** 8 weeks post-injury in the TIIA-PLGA SRMs group. Scale bar: 50 μm.

Nissl staining revealed that at 1 week post-surgery, the sham surgery group exhibited neurons of normal multi-polar morphology, with a centrally located nucleus and clear Nissl bodies (Figure 5A). In the SCI group, a large number of neurons died, causing vacuoles to form; the Nissl bodies either dissolved and disappeared or remained residually in the vacuoles, while the nucleus were not visible (Figure 5B). The STS group showed some swollen neuronal cell bodies, with rounded and blunt protrusions on the surface, appearing nearly circular; the Nissl bodies were partially dissolved, forming a ring shape, while the nucleoli were either missing or located at the edge of the nucleus. Occasionally, neurons with normal morphology were observed (Figure 5C). The TIIA-PLGA SRMs group exhibited some normal neurons at 1 week, with morphology similar to that of the sham surgery group. Occasionally, damaged neurons and vacuoles were observed (Figure 5D). At 8 weeks post-surgery, the sham surgery group showed similar features to 1 week post-surgery (Figure 5E). In the SCI group, neuronal death increased due to secondary damage, and vacuoles were denser and more widespread than at 1 week, with fragments of Nissl bodies or nucleoli visible in some vacuoles (Figure 5F). The STS group at 8 weeks showed a transition of damaged neurons from death to survival, with a decrease in the extent and density of vacuoles compared with the SCI group. A small number of normal neurons were visible (Figure 5G). The TIIA-PLGA SRMs group at 8 weeks showed more surviving damaged neurons compared with the STS group, with a larger number of normal neurons and fewer vacuoles (Figure 5H).

### 3.6 Intrathecal administration of TIIA-PLGA SRMs alleviates neuroinflammatory response by modulating microglial phenotypic polarization after SCI

**FIGURE 6:**
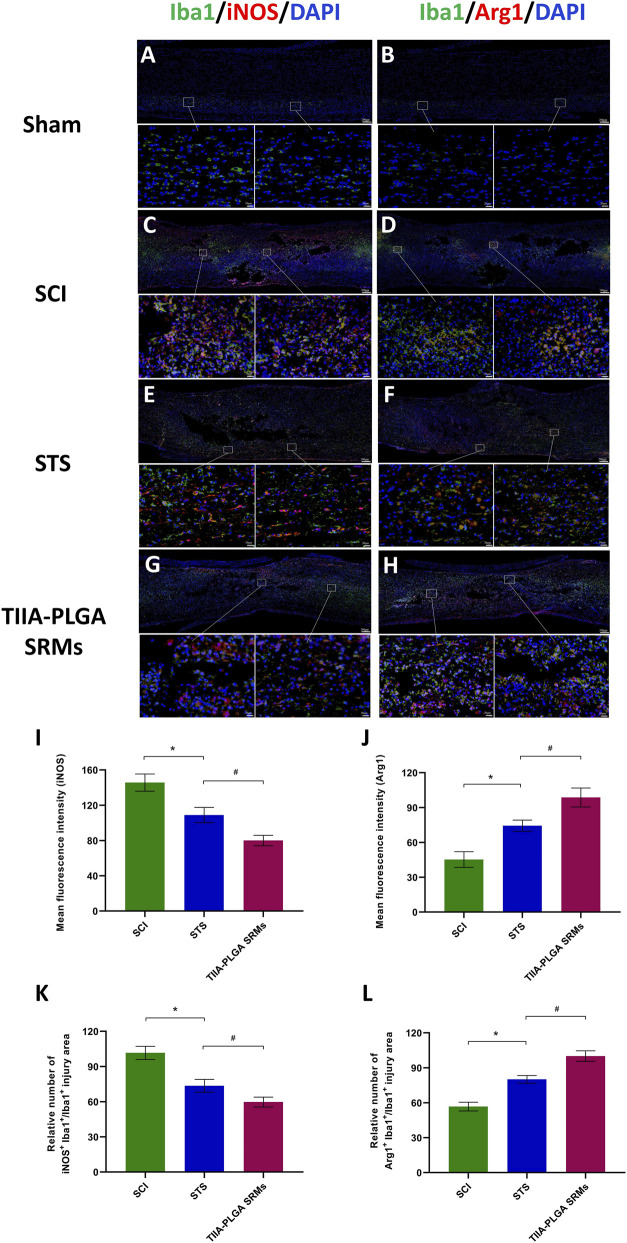
Immunofluorescence staining results of spinal cord tissue at the injury site in rats 1 week post-SCI. **(A)** M1-type microglia staining in the sham surgery group; **(B)** M2-type microglia staining in the sham surgery group; **(C)** M1-type microglia staining in the SCI group; **(D)** M2-type microglia staining in the SCI group; **(E)** M1-type microglia staining in the STS group; **(F)** M2-type microglia staining in the STS group; **(G)** M1-type microglia staining in the TIIA-PLGA SRMs group; **(H)** M2-type microglia staining in the TIIA-PLGA SRMs group. Scale bar = 200 μm in spinal cord overall view, and scale bar = 20 μm in details. **(I)** Quantitative analysis of iNOS fluorescence intensity; **(J)** Quantitative analysis of Arg1 fluorescence intensity; **(K)** Count of M1-type microglia; **(L)** Count of M2-type microglia; Data are expressed as the means ± S.D. (*n* = 6 per group). *P < 0.05, vs. SCI group; #*P* < 0.05 vs. STS group.

We investigated whether TIIA-PLGA SRMs alleviate neuroinflammatory response by modulating microglial phenotypic polarization 7 days after SCI using immunofluorescence, Western blotting, and ELISA. Immunofluorescence staining showed that in the sham surgery group, spinal tissues contained scattered green fluorescent-labeled (Iba1^+^ + iNOS^−^/Arg1^-^ + DAPI^+^) unactivated microglia (M0 type) (Figures 6A,B). In the SCI, STS, and TIIA-PLGA SRMs groups, red-green fluorescence co-labeled (Iba1^+^ + iNOS^+^/Arg1^+^ + DAPI^+^) positive cells (activated M1/M2 type microglia) were observed, and were distributed mainly in the SCI segment and its adjacent segments (Figure 6C‒H). Compared with the SCI group, the STS group demonstrated a reduction in both the fluorescence intensity and the number of M1-type microglia (Iba1^+^ + iNOS^+^ + DAPI^+^), alongside an increase in both the fluorescence intensity and the number of M2-type microglia (Iba1^+^ + Arg1^+^ + DAPI^+^) (*P* < 0.05). Additionally, compared with the STS group, the TIIA-PLGA SRMs group exhibited a significantly greater decrease in both the fluorescence intensity and the number of M1-type microglia, as well as a significant increase in both the fluorescence intensity and the number of M2-type microglia (*P* < 0.05) (Figure 6I‒L).

**FIGURE 7:**
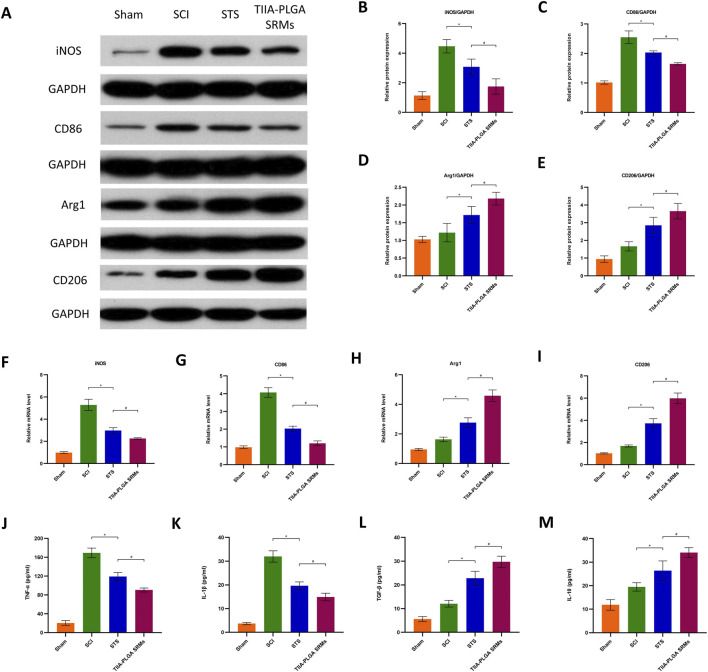
Microglial phenotypic polarization and neuroinflammatory response 1 week post-SCI in rats. **(A‒E)** Western blot and quantitative analysis of microglial phenotypic polarization markers (iNOS, CD86, Arg1, and CD206). Data are expressed as the means ± S.D. (*n* = 3 per group). *P < 0.05, vs. SCI group; #*P* < 0.05 vs. STS group. **(F‒I)** Quantitative analysis of iNOS, CD86, Arg1, and CD206 mRNA levels. Data are expressed as the means ± S.D. (*n* = 3 per group). *P < 0.05, vs. SCI group; #*P* < 0.05 vs. STS group. **(J‒M)** ELISA detection of levels of pro-inflammatory factors (TNF-α and IL-1β) and anti-inflammatory factors (TGF-β and IL-10) in spinal cord tissues of rats with spinal cord injury; Data are expressed as the means ± S.D. (*n* = 3 per group). *P < 0.05, vs. SCI group; #*P* < 0.05 vs. STS group.

Microglia in rat spinal cord tissue are activated after SCI. Western blotting showed that the expression levels of M1-type microglia markers (iNOS and CD86) and M2-type microglia markers (Arg1 and CD206) were significantly higher in all experimental groups compared with the sham surgery group (*P* < 0.05). Meanwhile, the expression levels of M1-type microglia markers in the STS group were significantly lower than those in the SCI group, while the expression levels of M2-type microglia markers in the STS group were significantly higher than those in the SCI group (*P* < 0.05). In addition, when compared with the STS group, the TIIA-PLGA SRMs group showed a significantly greater decrease in the expression of M1-type microglia markers and a significantly greater increase in the expression of M2-type microglia markers (*P* < 0.05) (Figure 7A‒E). Consistently, RT-PCR showed a similar trend (Figure 7F‒I).

SCI causes disruption of the microenvironment in the rat spinal cord, thereby disrupting the anti-inflammatory/pro-inflammatory balance. ELISA showed that the levels of pro-inflammatory factors (TNF-α and IL-1β) and anti-inflammatory factors (IL-10 and TGF-β) were significantly higher in all experimental groups compared with the sham surgery group (*P* < 0.05). Meanwhile, the levels of pro-inflammatory factors in the STS group were significantly lower than those in the SCI group, while the levels of anti-inflammatory factors in the STS group were significantly higher than those in the SCI group. In addition, when compared with the STS group, the TIIA-PLGA SRMs group showed a significantly greater decrease in the levels of pro-inflammatory factors and a significantly greater increase in the levels of anti-inflammatory factors (*P* < 0.05) (Figure 7J‒M). These results indicate that TIIA-PLGA SRMs can promote the early polarization of microglia towards the anti-inflammatory M2 type after SCI and inhibit polarization towards the pro-inflammatory M1 type. This confirms that intrathecal administration of TIIA-PLGA SRMs alleviates neuroinflammatory response by modulating microglial phenotypic polarization after SCI, and this treatment effect is superior to intraperitoneal injection of STS.

### 3.7 Intrathecal administration of TIIA-PLGA SRMs may modulate microglial phenotypic polarization after SCI by suppressing the notch signaling pathway

**FIGURE 8:**
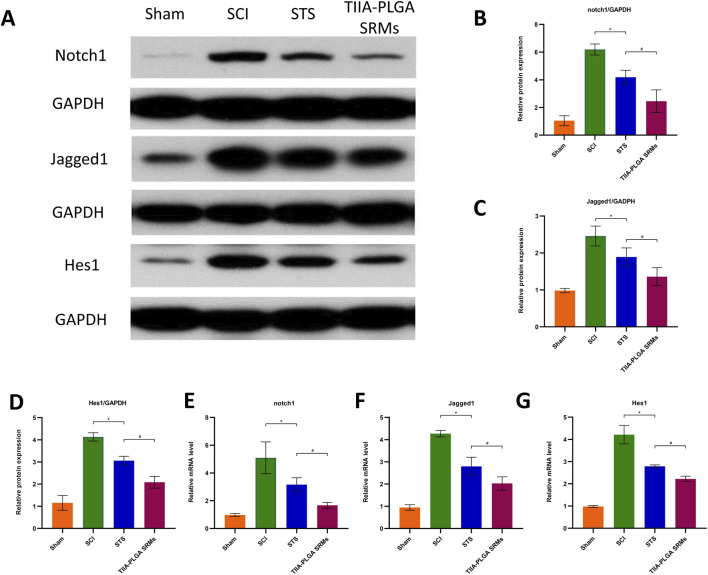
Potential mechanism of TIIA-PLGA SRMs modulating microglial phenotypic polarization from M1 to M2 after 1 week Post-SCI. **(A)** Western blot analysis of the Notch signaling pathway (Notch1, Jagged1, and Hes1). **(B‒D)** Quantitative assessment of Notch signaling pathway proteins (Notch1, Jagged1, and Hes1). Data are expressed as the means ± S.D. (*n* = 3 per group). *P < 0.05, vs. SCI group; #*P* < 0.05 vs. STS group. **(E–G)** Quantitative analysis of Notch1, Jagged1, and Hes1 mRNA levels. Data are expressed as the means ± S.D. (*n* = 3 per group). *P < 0.05, vs. SCI group; #*P* < 0.05 vs. STS group.

The Notch signaling pathway may be involved in the cellular signal transduction process through which intrathecal administration of TIIA-PLGA SRMs exerts its modulatory effect on microglial phenotypic polarization after SCI. Therefore, at 7 days post-SCI, we assessed the activation status of the Notch signaling pathway in the rat spinal cord in each group using Western blotting. The results showed that the expression of Notch signaling pathway-related proteins (Notch1, Jagged1, Hes1) was activated in the spinal cords of SCI, STS, and TIIA-PLGA SRMs groups, and the expression of these proteins was higher than that in the sham surgery group. The levels of Notch signaling pathway-related proteins in the STS group were significantly lower than those in the SCI group (*P* < 0.05). In addition, when compared with the STS group, the TIIA-PLGA SRMs group had greater significant decrease in the levels of Notch signaling pathway-related proteins (*P* < 0.05) (Figure 8A‒D). Similarly, RT-PCR showed a similar trend (Figure 8E‒G). These results suggest that intrathecal administration of TIIA-PLGA SRMs may suppress the Notch signaling pathway, thereby modulating microglial phenotypic polarization after SCI, and this effect is superior to that of the STS group.

## 4 Discussion

SCI is a highly challenging clinical problem due to its complex and dynamic pathological processes (Bie et al., 2021; Poulen et al., 2021). To date, there are no effective therapeutic strategies that are able to overcome these challenges completely. In this study, we found that intrathecal administration of TIIA-PLGA SRMs may alleviate neuroinflammatory response by suppressing the Notch signaling pathway after SCI, thereby promoting motor function recovery and improving neuropathic pain in rats.

An increasing body of research suggests that microglial cells play a significant role in the pathophysiological changes independently or in coordination with other neuroglial cells, immune cells, and inflammatory cells after SCI, making them a potential breakthrough in basic and clinical research on SCI [(St-Pierre et al., 2023; Xu et al., 2022; Xu A. H. et al., 2023)]. Many researchers believe that inhibiting the excessive immune/inflammatory response after SCI may slow down the cascade reactions in secondary injury. This can help control the expansion of the injury range while preserving the function of the remaining neural tissue as much as possible, providing a suitable microenvironment for future neural repair and regeneration (Zhu et al., 2024; Liu et al., 2024). As one of the three major regulatory systems in the body (immune system, nervous system, endocrine system), the immune system plays a crucial role in clearing necrotic tissues and harmful substances, repairing damages, and maintaining stability of the internal environment and physiological balance (He et al., 2023; Valido et al., 2023). Currently, microglial cells are considered the main resident immune cells in the spinal cord and play an important part in constructing the spinal cord immune network (Poppell et al., 2023). Microglial cells originate from early embryonic red-marrow progenitor cells (microglial precursor cells) produced within the yolk sac and settle in the developing spinal cord before the formation of the BSCB. They subsequently mature in the unique neural microenvironment constituted by the BSCB, maintaining their own numbers. Microglial cells differ from macrophages in terms of cell origin, gene expression, and biological functions (Thion et al., 2018; Zhang L. et al., 2021). After SCI, microglia undergo activation into M1 and M2 phenotypes, which are associated with pro-inflammatory and anti-inflammatory roles, respectively. Moderating the polarization of microglia holds potential for enhancing the inflammatory microenvironment of the spinal cord and facilitating SCI repair (Akhmetzyanova et al., 2023; Brockie et al., 2021).

The Notch signaling pathway, a highly conserved signal transduction molecule in evolution, is involved in the regulation of cellular processes such as migration, proliferation, differentiation, activation, and apoptosis (Medina et al., 2023). After SCI, the Notch signaling pathway is excessively activated, and is associated with pathological conditions such as inflammatory cell infiltration, upregulation of pro-inflammatory cytokine expression, restricted neuronal regeneration and differentiation, difficulty in microvascular and axonal regeneration, proliferation of astrocytes, and scar formation (Jacobs and Huang, 2021; Chen et al., 2023). Studies have also shown that the Notch signaling pathway is involved in the regulation of microglial phenotypic polarization. Specifically, activation of the Notch signaling pathway promotes M1 polarization of microglia, leading to the release of large amounts of pro-inflammatory cytokines (e.g., TNF-α, IL-1β, IL-6), which exacerbate local inflammation and impede neuronal regeneration and repair. In contrast, inhibition of the Notch signaling pathway can suppress M1 polarization of microglia while promoting M2 polarization (Jian et al., 2020; Liao et al., 2020; Wu et al., 2019). Additionally, other studies suggest that the effects of inhibiting the Notch signaling pathway on microglial polarization phenotypes are irreversible. Normally, lipopolysaccharide stimulation can induce the transformation of M2 microglia into the M1 phenotype, but this phenomenon does not occur in M2 microglia previously treated with Notch inhibitors (Liu et al., 2012). Thus, blocking the Notch signaling pathway can create a “memory” effect in microglial polarization phenotypes. In the context of SCI treatment, inhibiting the excessive activation of the Notch signaling pathway may help regulate the long-term balance of M1/M2 polarization of microglia, maintaining a state conducive to injury recovery, offering promising therapeutic potential.

TIIA, a monomer derived from traditional Chinese medicine known for its significant anti-inflammatory and neuroprotective properties, has been demonstrated in previous studies to promote neurological recovery after SCI. Luo et al. (2022) found that TIIA facilitates the repair of damaged microvasculature and the BSCB following SCI, thereby decreasing BSCB permeability and enhancing neurological function in SCI rats. Our previous research confirmed that TIIA mitigates inflammation and edema in bladder tissue after SCI, leading to improved urodynamic parameters and promoting recovery from lower urinary tract dysfunction (Yang et al., 2017). Additionally, Yin et al. (2012) reported that TIIA exerts an inhibitory effect on apoptosis in spinal cord tissue post-SCI and attenuates neuroinflammation, although the underlying mechanisms remain unclear. Some studies have confirmed the regulatory effect of TIIA on the Notch signaling pathway. The *in vitro* experiment completed by Zhao et al. (2025) demonstrated that TIIA can inhibit the expression of Notch signaling pathway-related proteins (Notch1, Hes1), thereby reducing the M1 polarization phenotype of macrophages in the atherosclerosis model. Zhong et al. (2025) confirmed through *in vivo* and *in vitro* experiments that TIIA can suppress the excessive activation of the Notch signaling pathway, thereby promoting the proliferation and differentiation of endogenous neural stem cells to repair SCI in rats. This study demonstrates that the sulfonated derivative of TIIA, known as STS, modulates microglial polarization through the Notch signaling pathway, thereby mitigating inflammatory responses surrounding the injured spinal cord after SCI. However, the inherent limitations of TIIA’s poor bioavailability and STS’s instability necessitate the exploration of innovative drug delivery systems or formulations for TIIA. The objective is to increase TIIA’s solubility in body fluids and improve its bioavailability at the injury site (Rao et al., 2022). In this study, we used PLGA SRMs as carriers for TIIA and prepared them using the emulsion electrospray method. Scanning electron microscopy observation showed that the prepared TIIA-PLGA SRMs were spherical with regular morphology and smooth surface, and were of uniform particle size. At the same time, *in vitro* release experiments indicated that SRMs could effectively release TIIA, with a stable release process and no apparent burst effect, demonstrating good sustained release performance. This laid the foundation for subsequent animal experiments on SCI intervention.

SCI has not been fully overcome by modern medicine, and most research is still in the stage of basic medical research, mainly based on animal experiments. The conventional routes of administration for animal SCI models are primarily gastric administration, intraperitoneal injection, and tail vein injection. However, due to the barrier function of the BSCB, the transport of most drug molecules from the plasma to the injured spinal cord tissue is restricted, leading to a significantly reduced drug delivery efficiency (Yang et al., 2017; Hou et al., 2022; Shen et al., 2023). The secondary inflammatory response and vascular dysfunction resulting from SCI induce a temporary increase in the permeability of the BSCB, which persists during the initial 7 days post-injury (Xie et al., 2023). However, this pathological and transient increase in permeability gradually normalizes over time, failing to satisfy the sustained therapeutic requirements for SCI. Consequently, conventional administration methods, such as gavage or intravenous injection, demonstrated limited efficacy in the pharmacological management of SCI. Intrathecal administration is an effective and practical route of administration that can directly deliver drugs into the subarachnoid space to reach the lesion site of the spinal cord, thereby avoiding limitations of the BSCB on drug transport from the blood circulation, and also, the first-pass elimination of gastrointestinal administration. This approach reduces the cost of drug administration, improves drug delivery efficiency, and avoids biosafety issues associated with intravenous administration of special drugs such as stem cells (Yao et al., 2022). Therefore, intrathecal administration has been increasingly used in SCI experimental interventions in recent years. However, there are still issues such as the small single injection volume (less than 30 μL) and the need for long-term placement of intrathecal catheters or multiple percutaneous puncture administrations in some experiments (Bang et al., 2018). The placement of intrathecal catheters may cause damage to the central nervous system, and the risk of catheter blockage or detachment exists after catheter placement (Liu et al., 2014). Percutaneous intrathecal injection is challenging to perform and may lead to nerve damage (Zheng et al., 2019). Furthermore, our preliminary experiments revealed that the restricted subarachnoid space in conventional intrathecal administration presented substantial challenges for executing a single large-dose intrathecal injection. This limitation may impede the effective delivery of therapeutics, particularly when larger doses are required for optimal therapeutic effect. In contrast, intrathecal injection via the atlanto-occipital membrane allows for the safe administration of a larger volume of TIIA-SRMs in a single injection. This method not only overcomes the spatial limitation associated with conventional techniques but also gives full play to the sustained-release properties of TIIA-SRMs, thereby maximizing their therapeutic efficacy over an extended period. Therefore, in this study, to fully exploit the pharmacological advantages of TIIA-PLGA SRMs (long-term sustained slow release, reduced number of administrations and total dose, increased bioavailability), and to avoid the barrier effect of BSCB on drugs, we used intrathecal injection through the atlanto-occipital membrane as the administration route in rats after SCI.

To investigate the impact and associated mechanisms of intrathecal injection of TIIA-PLGA SRMs on microglial polarization in SCI rats, we established an incomplete SCI model using the NYU impactor. After successful model establishment, we examined the markers of M1/M2 microglia in rats with SCI through immunofluorescence staining, RT-PCR and Western blot techniques. Additionally, the levels of neuroinflammatory response after SCI were assessed using ELISA. The results showed that TIIA-PLGA SRMs could promote the polarization of microglia towards the anti-inflammatory M2 phenotype in the early stage of SCI, while inhibiting the polarization towards the pro-inflammatory M1 phenotype. This confirmed that intrathecal administration of TIIA-PLGA SRMs could alleviate neuroinflammation by modulating the polarization of microglial cells after SCI, and its effectiveness was superior to intraperitoneal injection of STS. Furthermore, comprehensive analysis of behavioral, neuroconduction function, and histopathological examinations confirmed the efficacy of intrathecal injection of TIIA-PLGA SRMs in treating SCI, and its efficacy was superior to traditional intraperitoneal injection of STS. The above results confirm that in the experimental intervention of SCI with TIIA in rats, the innovative treatment concept of “sustained-release formulation + intrathecal administration” (TIIA-PLGA SRMs intrathecal injection) demonstrates certain advantages over the conventional concept (STS intraperitoneal injection). This may be attributed to the fact that intrathecal injection via the atlanto-occipital membrane allows a single administration of a relatively large dose of TIIA-PLGA SRMs into the rat’s intrathecal space. By retaining the microspheres within the intrathecal space and gradually releasing TIIA, this method achieves prolonged targeted therapeutic effects for SCI, thereby reducing administration frequency, enhancing bioavailability, and circumventing the hindrance posed by the BSCB to conventional drug molecules. In addition, Western blot analysis and RT-PCR was performed to assess the activation of the Notch signaling pathway in the spinal cords of rats in the different groups after SCI. The results indicated that intrathecal administration of TIIA-PLGA SRMs could also exert an inhibitory effect on the Notch signaling pathway. Therefore, we hypothesize that their regulatory effect on microglial polarization may be achieved through the inhibition of the Notch signaling pathway.

This study has several limitations. First, the absence of an STS intrathecal injection group limits the direct comparison of the effects of different administration routes. While we considered that STS is primarily administered intravenously in clinical settings, and that intraperitoneal injection could achieve a somewhat similar effect, the lack of experimental design for STS intrathecal injection prevents us from fully evaluating its relative efficacy and safety in treatment. Second, this study does not provide evidence that TIIA-PLGA SRMs can remain in the lesion site for an extended period and sustained release TIIA. Although we aimed to enhance the local concentration of the drug in the injury area through intrathecal injection, the lack of direct observation of the retention of microspheres in the cerebrospinal fluid over time limits our ability to confirm their sustained release effects. In response to these issues, we are conducting *in vivo* pharmacokinetic studies of TIIA-PLGA SRMs after intrathecal administration, observing their retention and degradation timelines as well as *in vivo* release behavior. PLGA is regarded as a medical polymer material with good biodegradability and biocompatibility, and is applied as a high-quality drug carrier in basic research (Tang et al., 2026). However, the potential inflammatory response during intrathecal administration has not been clearly identified, so the clinical safety of this intrathecal delivery system still needs to be considered. Third, In this study, there were some limitations in the evaluation methods of pathological morphology results such as HE and Nissl staining. For instance, the pathological morphology score based on HE staining used five integers ranging from 0 to 4 to evaluate pathological morphological changes of the injured spinal cord. The evaluation method was relatively coarse and subjective, and the infiltrating immune or inflammatory cells were difficult to identify. In subsequent studies, more detailed, objective, and repeatable pathological morphology evaluation methods should be explored. Next, this study did not conduct *in vitro* experiments targeting microglia or intervention experiments with Notch signaling pathway agonists, which restricts our further understanding of the mechanism of the Notch signaling pathway involvement in the treatment of SCI with TIIA-PLGA SRMs and its roles in regulating microglial phenotypic polarization. Finally, our study used Iba1 as a standard microglia marker, but Iba1 is expressed by both resident microglia and infiltrating monocyte-derived macrophages after SCI. In the future, specific microglia markers such as TMEM119 or P2RY12 should be used to distinguish these populations and reduce the interference from macrophages in the results.

## 5 Conclusion

Intrathecal injection through the atlanto-occipital membrane of TIIA-PLGA SRMs has been demonstrated to effectively promote the early shift of microglial phenotype from M1 to M2 in rats with SCI. This modulation alleviates neuroinflammatory responses, consequently improving motor dysfunction and neuropathic pain following SCI. Notably, the efficacy of TIIA-PLGA SRMs surpasses that of traditional intraperitoneal injection of STS. Furthermore, the regulatory role of TIIA-PLGA SRMs in microglial polarization after SCI is likely associated with the inhibition of the Notch signaling pathway. In conclusion, TIIA-PLGA SRMs, administered via intrathecal injection through the atlanto-occipital membrane, offer a promising therapeutic approach for mitigating the consequences of SCI, with potential implications for future clinical applications.

## Data Availability

The original contributions presented in the study are included in the article/Supplementary Material, further inquiries can be directed to the corresponding authors.
