# Supplementary figures and images for: Regulation of microglial polarization via notch signaling pathway by intrathecal administration of tanshinone IIA-PLGA sustained-release microspheres to promote neurological recovery after spinal cord injury
Source: Front Bioeng Biotechnol. 2026 Jun 5;14:1784592. doi: 10.3389/fbioe.2026.1784592 (PMC13279315; doi:10.3389/fbioe.2026.1784592)

**Western Blot raw data**


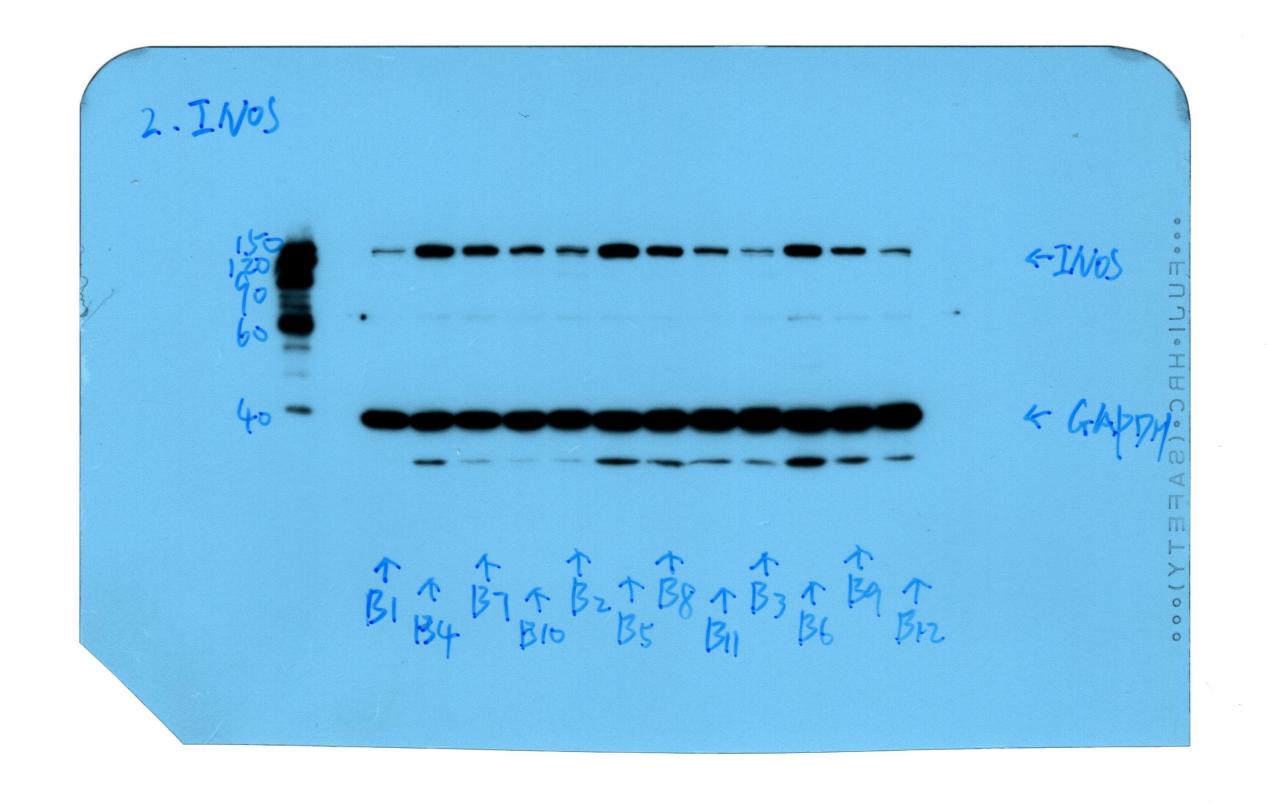


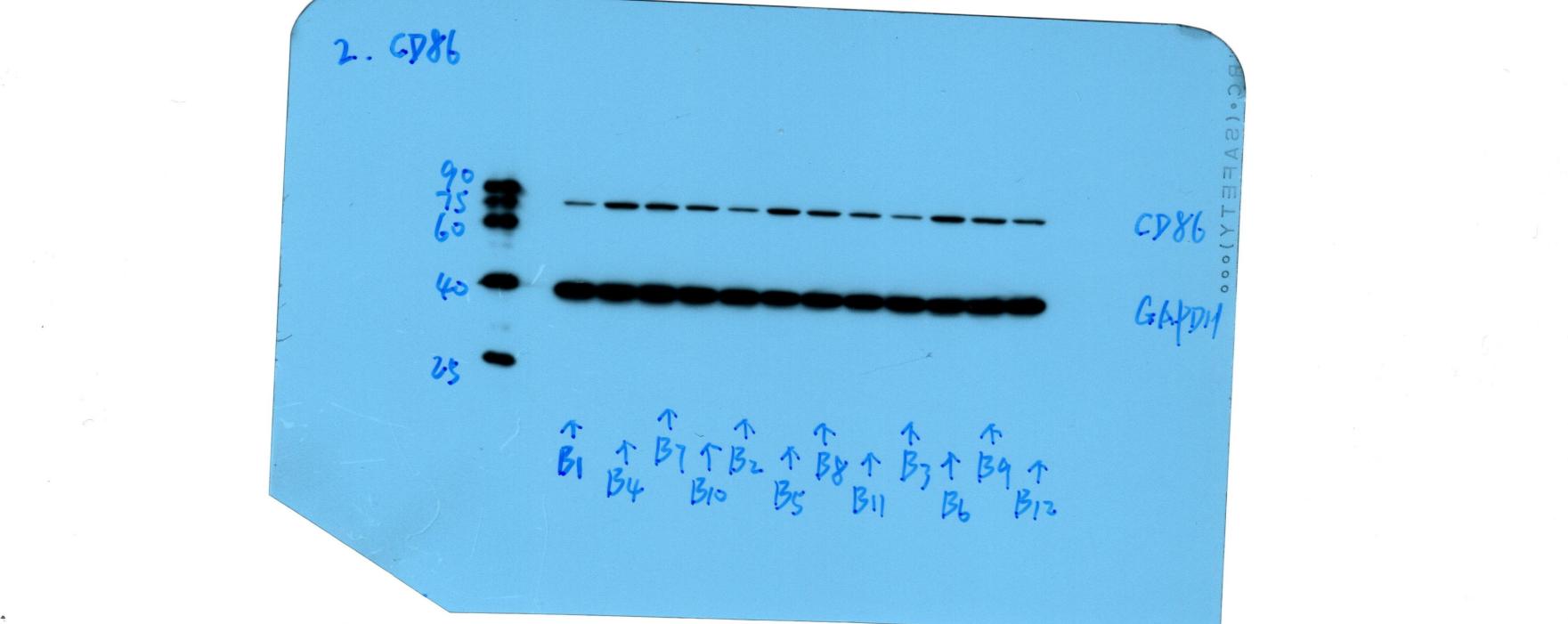


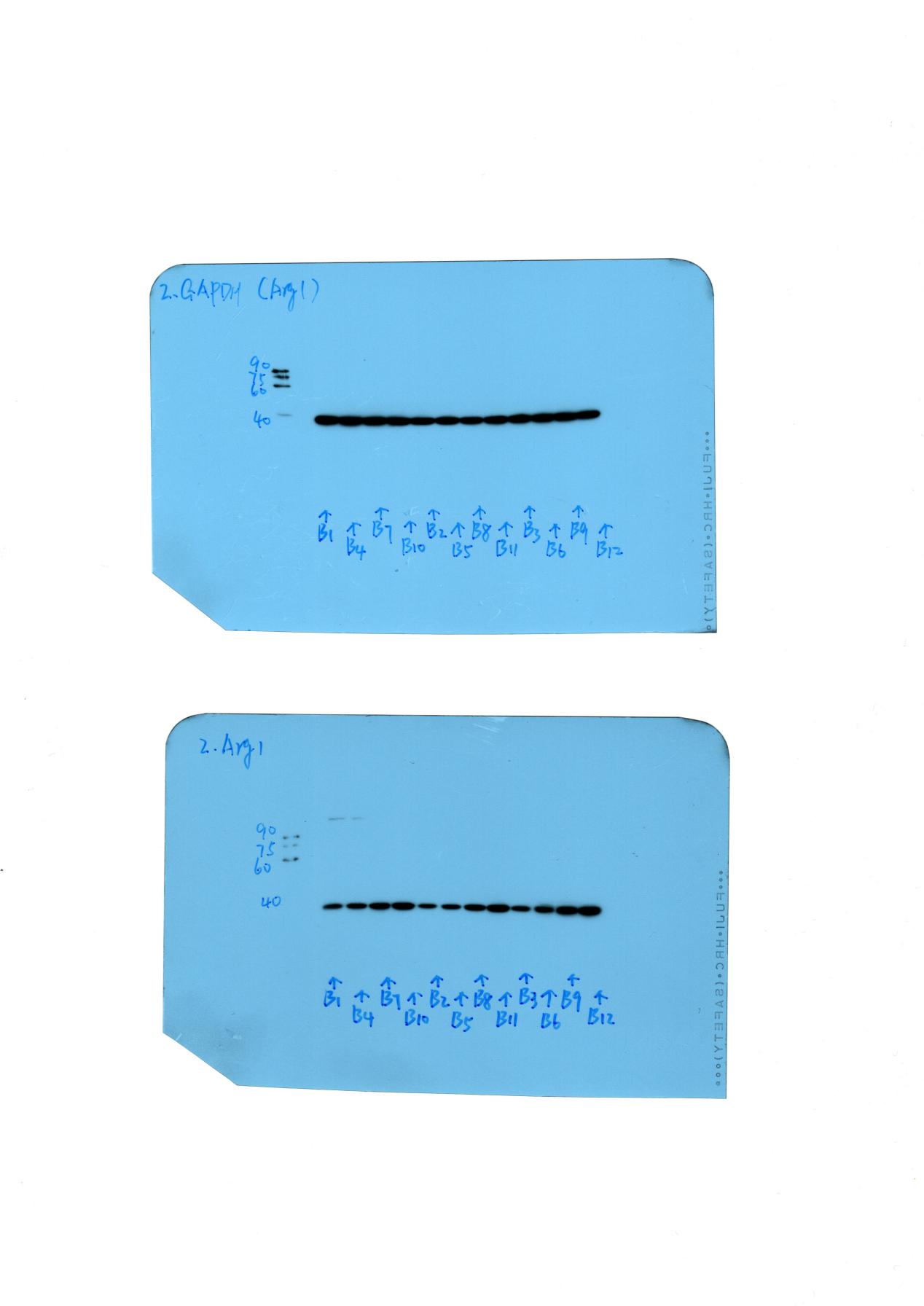


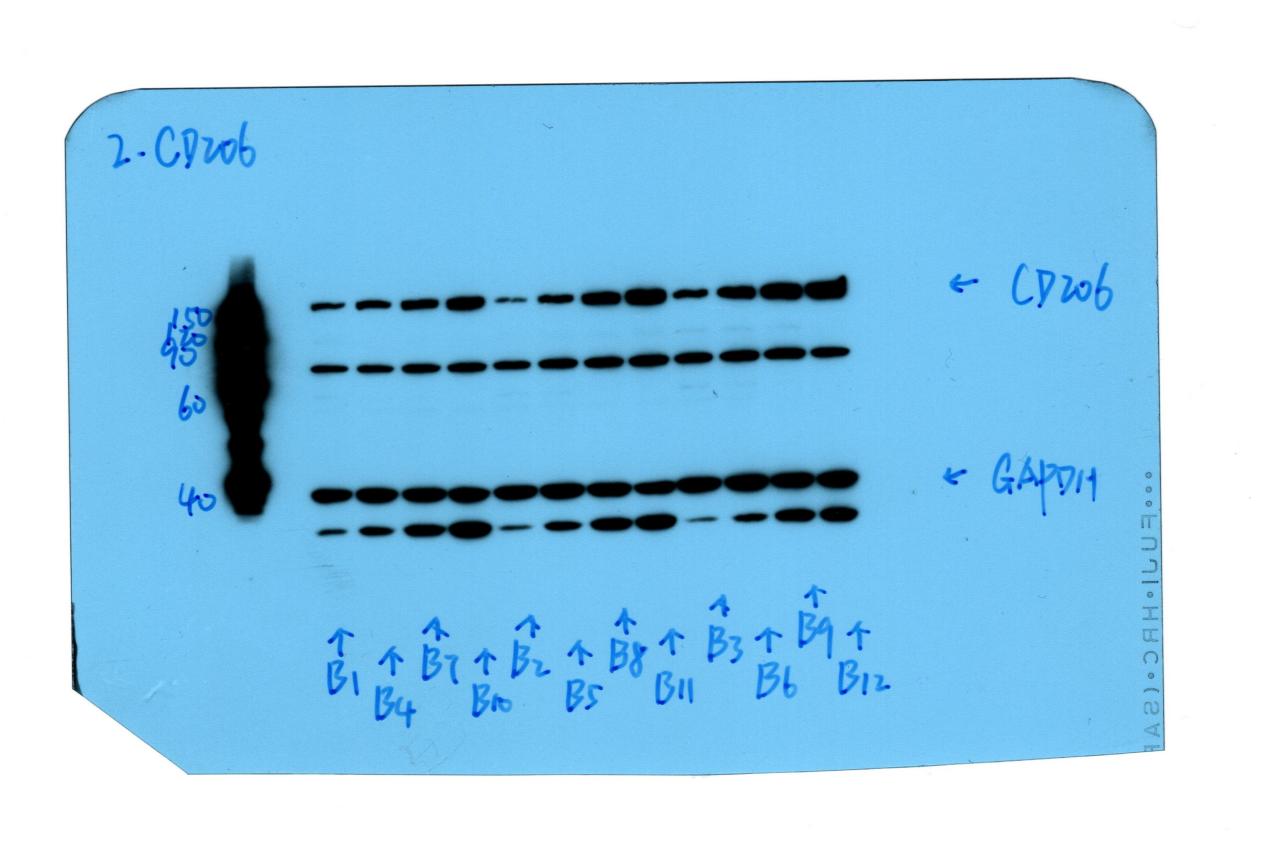


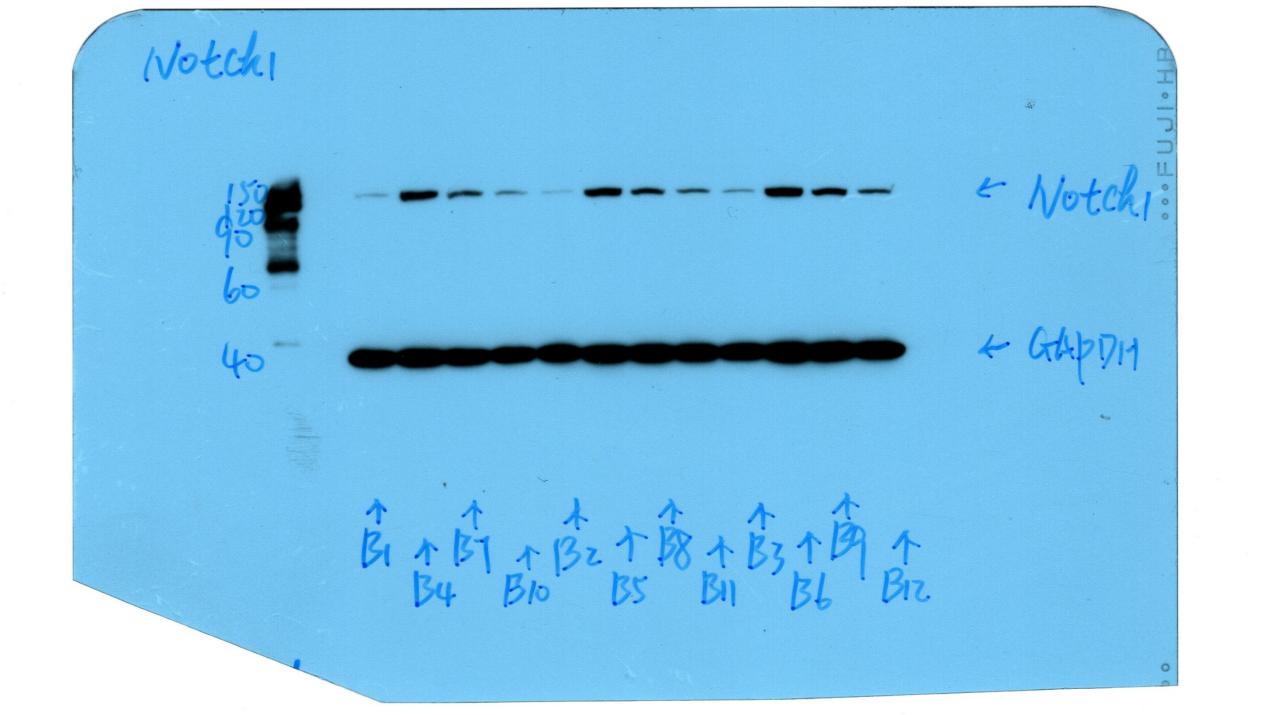


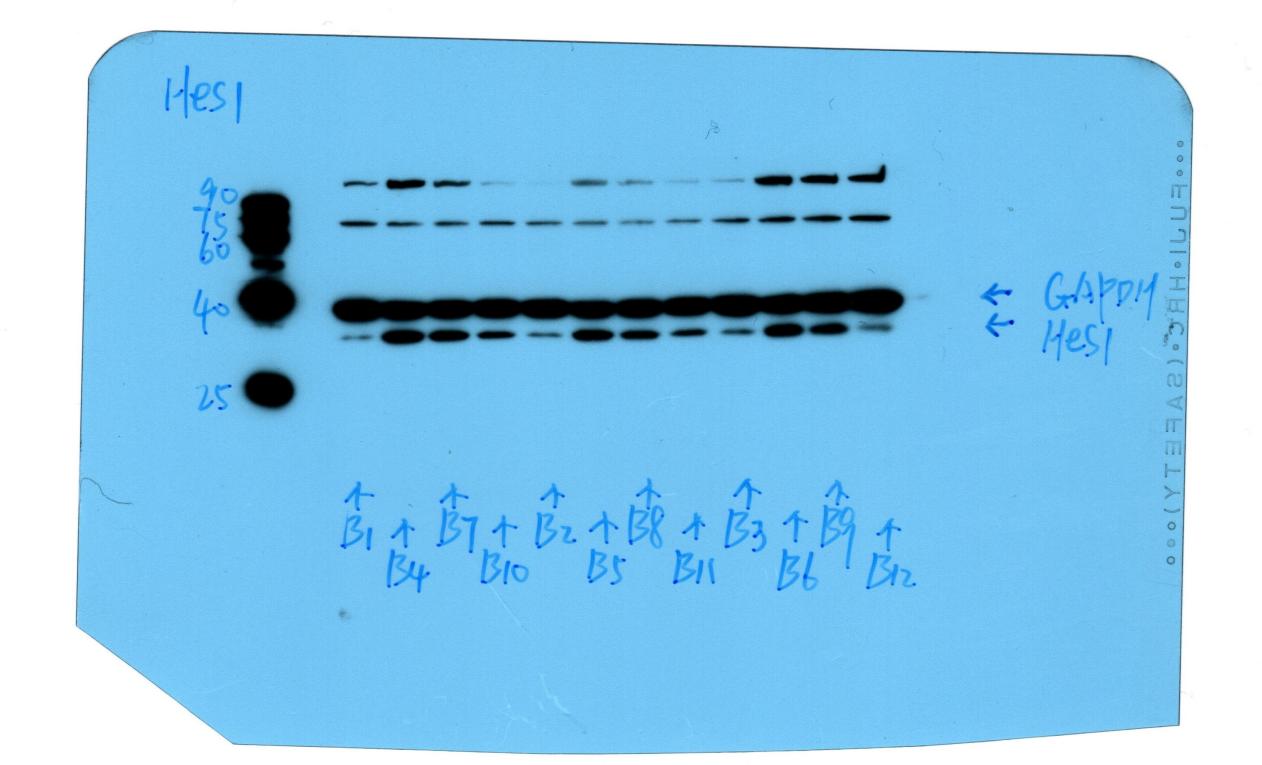


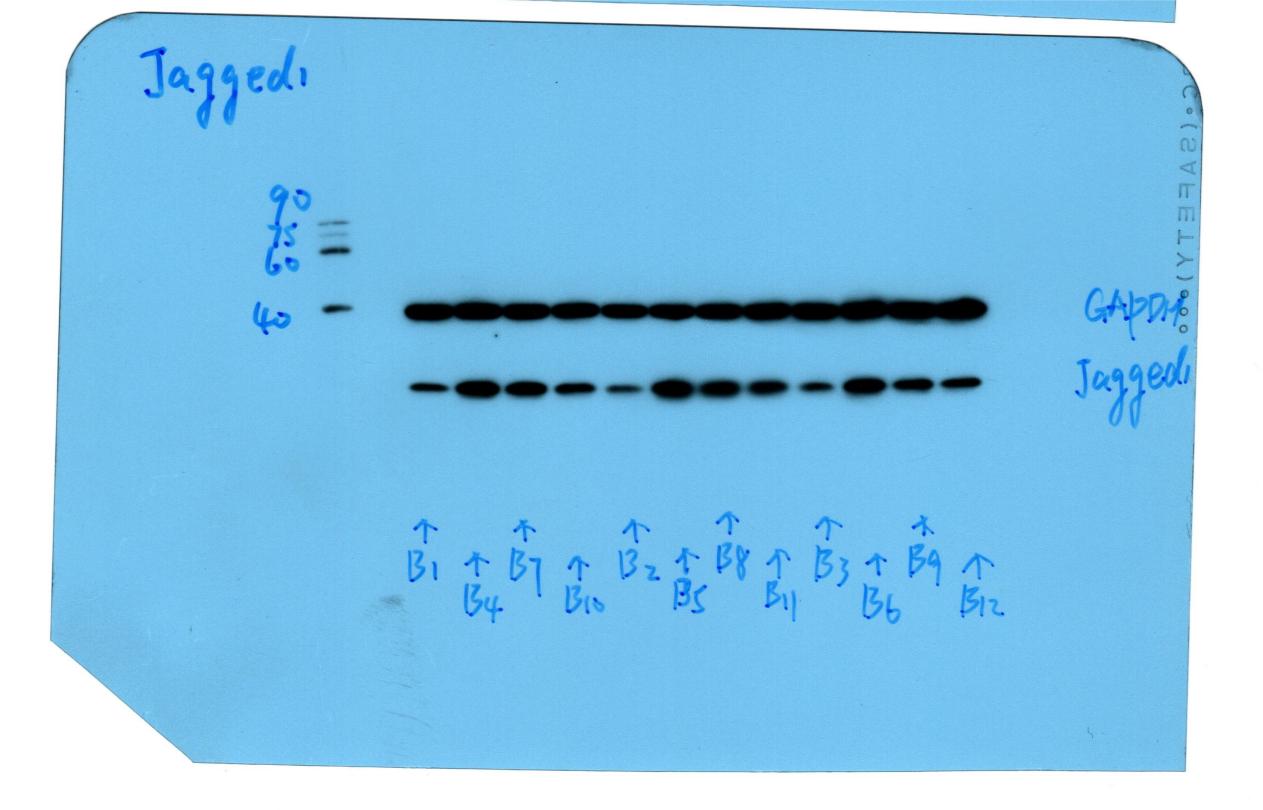

Supplement: Supplementary file 3 [file Table1.docx]
